# Durable 3D murine ex vivo retina glaucoma models for optical coherence tomography

**DOI:** 10.1364/BOE.494271

**Published:** 2023-08-02

**Authors:** Álvaro Barroso, Steffi Ketelhut, Gerburg Nettels-Hackert, Peter Heiduschka, Rocío del Amor, Valery Naranjo, Björn Kemper, Jürgen Schnekenburger

**Affiliations:** 1 Biomedical Technology Center of the Medical Faculty, University of Muenster, Mendelstr. 17, D-48149 Muenster, Germany; 2 Department of Ophthalmology of the Medical Faculty, University of Muenster, Domagkstr. 15, D-48149 Muenster, Germany; 3 Instituto Universitario de Investigación en Tecnología Centrada en el Ser Humano, Universitat Politècnica de València, Valencia, Spain

## Abstract

Durable and standardized phantoms with optical properties similar to native healthy and disease-like biological tissues are essential tools for the development, performance testing, calibration and comparison of label-free high-resolution optical coherence tomography (HR-OCT) systems. Available phantoms are based on artificial materials and reflect thus only partially ocular properties. To address this limitation, we have performed investigations on the establishment of durable tissue phantoms from ex vivo mouse retina for enhanced reproduction of in vivo structure and complexity. In a proof-of-concept study, we explored the establishment of durable 3D models from dissected mouse eyes that reproduce the properties of normal retina structures and tissue with glaucoma-like layer thickness alterations. We explored different sectioning and preparation procedures for embedding normal and N-methyl-D-aspartate (NMDA)-treated mouse retina in transparent gel matrices and epoxy resins, to generate durable three-dimensional tissue models. Sample quality and reproducibility were quantified by thickness determination of the generated layered structures utilizing computer-assisted segmentation of OCT B-scans that were acquired with a commercial HR-OCT system at a central wavelength of 905 nm and analyzed with custom build software. Our results show that the generated 3D models feature thin biological layers close to current OCT resolution limits and glaucoma-like tissue alterations that are suitable for reliable HR-OCT performance characterization. The comparison of data from resin-embedded tissue with native murine retina in gels demonstrates that by utilization of appropriate preparation protocols, highly stable samples with layered structures equivalent to native tissues can be fabricated. The experimental data demonstrate our concept as a promising approach toward the fabrication of durable biological 3D models suitable for high-resolution OCT system performance characterization supporting the development of optimized instruments for ophthalmology applications.

## 1. Introduction

Since its development in the 1990’s [1], optical coherence tomography (OCT) has been successfully established in ophthalmology as a powerful tool for non-invasive label-free imaging of the retina layers of the eye [2,3]. Various further developments of the technology, such as Fourier-domain OCT, swept-source OCT and more recently polarization sensitive and spectroscopic imaging [4–13], have significantly improved its application in ophthalmology, e.g., for assisting the in vivo diagnosis of diseases such as macular degeneration, corneal pathologies, multiple sclerosis and glaucoma, to mention a few [14,15]. Glaucoma is an optic neuropathy characterized by progressive loss of retinal ganglion cells (RGC) and optic nerve damage that can result in visual field loss and irreversible blindness [16]. Since loss of RGC cannot be directly detected in clinical settings due to the limited axial resolution of current ophthalmologic OCT systems, applicants typically use OCT for the evaluation of structural changes in the optic nerve head and other retinal layers that are affected by glaucoma. In this context, the development of high-resolution optical coherence tomography (HR-OCT) systems featuring higher axial resolution in a range of few micrometers and below promise important improvements in glaucoma diagnosis by more precise thickness determination of the involved degenerated retina layers.

Together with the advancements of novel HR-OCT systems, also the design of advanced calibration standards that allow measurement comparability between HR-OCT systems is important [17], since state-of-the-art instruments can vary in system calibration, scanning protocols, and software, e.g., concerning segmentation algorithms, which e. g. may cause disparities in thickness measurements between different instruments [18–20]. To address these topics, significant efforts and progress have been made to create standardized materials that allow calibration of OCT systems and, thus, provide an approach for validation and proof of reproducibility between trials and different systems [17,21]. A common strategy relies on the fabrication of tissue-mimicking materials known as phantoms. Typically, they consist of a bulk material with embedded scattering particles and absorbers or refractive structures that allow tuning the retina-mimicking material properties including optical, texture, elasticity, or blood flow properties [22–30]. To simulate three-dimensional heterogeneities commonly seen in retina tissue, various phantoms have been reported made of micrometer-sized layers, e.g., based on silicone or polymers like polydimethylsiloxane (PDMS) [28,31]. To resemble the different cells that are present in biological tissue, the specific optical properties of each layer can be tuned by loading the layer material with different types of nanoparticles, e.g., barium sulfate or titanium dioxide [31–35]. However, the fabrication of retina-mimicking phantoms requires relatively high efforts, suitable materials, as well as special know-how and equipment, and up to date, no artificial retinal phantoms for characterization and performance testing with anatomical structures close to the high axial resolution of HR-OCT systems have been reported.

In parallel to mimicking retina structures by artificial material, more practical strategies were conducted. In these approaches the heterogeneous thickness and reflectivity of individual retinal layers were generated by utilization of native and diseased biological tissues [36]. In this context, we performed a study on the establishment of reliable and durable ex-vivo 3D tissue models for performance testing of HR OCT systems, such as designed for glaucoma diagnosis. Animal models for glaucoma research include in particular mammalian models, such as rat, mouse and monkey [37–42]. Due to their small dimensions (about 13% of the human eye), mouse models determine the requirements on maximum resolution in HR OCT [43,44]. Murine retina tissue comprises thin layers of different cell types and tiny anatomic structures such as the ganglion cell layer and the optic nerve head. Currently, only a few animal models are known that develop spontaneous glaucoma. For example, DAB/2J mice develop pigment dispersion glaucoma [45,46]. However, only high age animals show increased intraocular pressure (IOP) and considerable loss of RGC. Furthermore, large variations were observed between individual animals. In addition, several transgenic mice were created based on already discovered mutations in glaucoma patients, e.g., mutations in the genes MYOC/GLC1A, OPTN/GLC1E, and the *tryptophan-aspartic acid* repeat domain 36 (GLC1G) [47–52]. These animals have been found applicable in some studies but represent only certain aspects of glaucoma. Due to the limitations of the above-mentioned existing animal models, we explored an approach based on C57BL/6J mice that were treated with N-methyl-D-aspartate (NMDA) to cause glaucoma-like retina alterations. C57BL/6J mice were successfully established in earlier studies as a glaucoma model in which the production of the neurotransmitter glutamate is increased in the retina as in case of glaucoma, aggravating the course of the disease [53,54]. Out of the ionotropic receptors for glutamate, the NMDA receptor is involved in the degeneration of RGC via its excitatory action [55]. The binding of NMDA to its receptor leads to influx of calcium, potassium, and sodium ions, inducing cell death. In addition, NMDA may also activate microglial cells, further enhancing damage to retinal neurons [56]. Intravitreal injection of NMDA was established as a model for RGC death and used for glaucoma research [57–60]. Following this earlier research, we used healthy eyes and eyes injected with NMDA from C57BL/6J mice to simulate retina degradation by glaucoma. For the fabrication of suitable 3D models, we explored various preparation concepts, including different fixation procedures, tissue shapes and several transparent embedding media, such as agarose and resin. Comparative investigations with a commercial HR OCT system, utilizing subsequent segmentation with custom build software to quantify the upper retinal layer thicknesses of the fabricated samples, were performed to demonstrate the feasibility of the proposed concept for fabrication of durable 3D test standards suitable for OCT applications.

## 2. Materials and methods

### 2.1 Preparation of 3D models from mouse retina with glaucoma-like properties

**Fig. 1.**
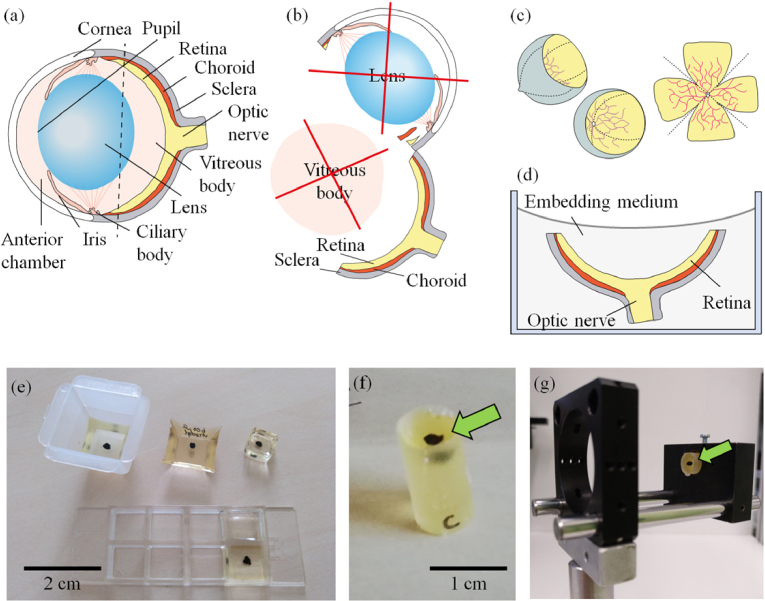
Illustration of the preparation procedures for generation of 3D retina models for OCT system testing from mouse eyes. (a) Sketch of the initially extracted mouse eye and (b) separation of the eyecup with removal of undesired structures. The cornea was removed from the eyeball by cutting in a circular manner using Vannas scissors. Then, lens and vitreous humor were taken out, while the retina, including the optical nerve head, remained within the eyecup. (c) Cutting scheme to achieve a clover leaf-like flat tissue from the eyecup. (d) Schematic of the retina prepared in the embedding medium. (e)-(f): Photos of representative 3D models from dissected mouse retina embedded in EPON resin and casted in (e) molds with different shapes and (f) a tube-shaped mold. (g) Photo of a tube-shaped 3D model constructed in an optical cage system. The green arrows in (f) and (g) indicate the sample visible as a black spot within the embedding medium.

C57BL/6J mice were chosen as the animal model for all experiments described in our study. To achieve degeneration of RGC, 1 µl of a 100 mM solution of NMDA in sterile phosphate buffered saline (PBS) was injected intravitreally into the eye of the mouse, thus injecting 100 nmol per eye. To perform the injection, the mice were anaesthetized transiently using isoflurane, in a mixture with oxygen at a concentration of 2 vol%. A drop of proparacaine was applied onto the eye to produce local anesthesia. The eye as well as the area around the eye were disinfected by betaisodona. Using colibri tweezers, the eyeball was pulled slightly out of the orbita. The conjunctiva was opened by Vannas scissors to expose the sclera. The sclera was then punctured by a sharp 30-gauge needle. Injection was performed using a 5 µl Hamilton syringe equipped with a 33-gauge needle. This needle was blunt to avoid mechanical damage to the retina. It was inserted through the hole into the vitreous, taking care not to damage the lens or the retina. The needle remained for further 3 to 4 seconds inside the eye to minimize reflux of the injected solution. The needle was finally retracted slowly out of the eye, and the eye was brought back to its normal position. Eyes injected with PBS or untreated eyes served as controls. Animals were killed and eyes were isolated one week after injection. For tissue fixation, the eyes were fixed either for 1 hour in 4% paraformaldehyde (PFA) or 2% glutaraldehyde solution in PBS for 4 hours. The eye then was pierced by a sharp scalpel at the limbus between the cornea and the sclera. The cornea was cut from the eyeball in a circular manner using Vannas scissors (Fig. 1(a)-(b)).

Two different shapes were investigated for the establishment of the 3D models, in the further text denoted as “eyecup concept” and “clover leaf concept”. In the first case, the retina remained in the eyecup and all other undesired structures were removed. In addition, the size of the eyecup was reduced by cutting the sclera and the retina in parallel to the limbus approximately at the equator of the eye. In the second case, the retina was isolated out of the eyecup by making four incisions from the periphery nearby to the optic nerve head and cutting the connection between the retina and the optic nerve (Fig. 1(c)). In this way, the retina can be disposed on planar surfaces, e.g., on glass object carrier slides, with the shape of a four-leaf clover. Finally, the sample was immersed in different embedding media including 1% phytagel (P8169, Sigma-Aldrich, St. Louis, USA), 1% agarose (Carl Roth, Karlsruhe, Germany; melting temperature ≤65.5 °C), EPON resin (Sigma-Aldrich, St. Louis, USA; curing time: 24 h at 60 °C) and Gedeo Crystal resin (GEMENOS, Cedex, France) (Fig. 1(d)). For embedding in resin media, samples were fixated in 1.25% glutaraldehyde and 2% formalin for several days, washed in tap water, and then dehydrated by ascending alcohol series, followed by subsequently incubation with propylenoxid. To achieve a defined position of the specimen below the resin-air interface the fixed and dehydrated retina was disposed on an initially semi solidified substrate in the mold and then poured with an additional amount of the substance. Figures 1(e)-(f) show exemplary samples embedded in EPON that were fabricated using molds with different shapes, including tube-shaped molds which facilitates later mounting of the 3D models in standard optical cage systems (Fig. 1 g). All preparation procedures on the fabrication and optimization on the 3D models as well the experimental investigations for their characterization were designed with the aim to minimize the number of animals used in the study.

### 2.2 OCT data acquisition

**Fig. 2.**
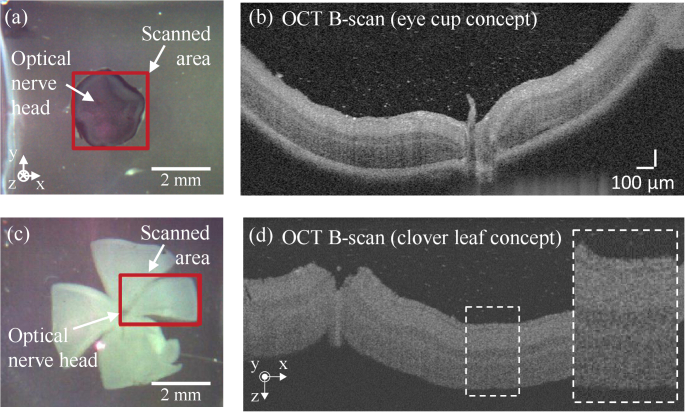
Illustration of OCT B-scan data acquisition for the example of PFA-fixated phytagel-embedded mouse retina prepared by (a)-(b) the eyecup and (c)-(d) the clover leaf concepts. (a), (c) Bright-field reflected light microscopy image and (b)-(d) exemplary B-scans from the acquired volume data.

For characterization of our tissue standards, OCT data were acquired using a high-resolution spectral domain OCT system (Thorlabs Ganymede Series, Thorlabs GmbH, Luebeck, Germany), which operates at a central wavelength of 905 nm with a 170-nm full-width at half-maximum (FWHM) spectral bandwidth. This OCT system is specified with an axial resolution of 3 µm in air, a depth-of-focus of 1.9 mm and the adjustable A-scan repetition rate was set to 36 kHz for all measurements in our study. OCT data were obtained utilizing the system’s software (ThorImage, version 5.2.0) using an objective lens with manufacturer-specified lateral resolution of 8 µm, working distance of 25.1 mm and field of view of 10 × 10 mm^2^ (LSM03-BB, Thorlabs GmbH, Luebeck, Germany). Optical path length detection in z-direction was calibrated during device production by the manufacturer by using a measuring tool consisting of two reflecting surfaces separated by a precisely defined distance. Furthermore, the OCT system featured a flexible adjustable probe head that allowed setting the focus at the RGC. We applied the 3D scanning mode of the OCT system’s software to acquire volume data of our developed 3D models as illustrated in Fig. 2 for the example of mouse retina prepared by the clover leaf concept (Fig. 1(c)). In particular, B-scans representing a scan area of approximately 5 mm^2^ per sample were acquired (e.g., see red rectangle in Fig. 2(a)) with up to 1400 A-line scans for each B-scan. The dispersion correction function of the acquisition software was applied in each measurement to obtain sharp B-scan images. The scanned area was centered approximately at the optical nerve head for samples prepared using the eyecup concept (see Fig. 2(a)). For samples prepared using the clover leaf concept, the scanned area was selected as depicted in the red rectangle in Fig. 2(c). Fig.2b and Fig. 2(d) show exemplary B-scans from the scanned areas depicted in Fig. 2(a) and Fig. 2(c), respectively. The distance between adjacent B-scans was set to 4 µm to enable subsequent image processing-based reduction of speckle noise by averaging of several consecutive B-scan images.

**Fig. 3.**
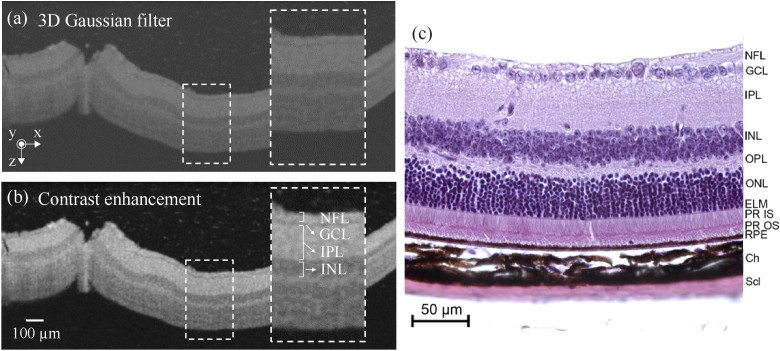
Illustration of image processing of OCT B-scan data for the example of phytagel-embedded mouse retina prepared by the clover leaf concept (see Fig. 2(c)–2(d)) (a) Removal of speckle noise in the B-scans by a 3D Gaussian filter and (b) contrast enhancement by applying a modified linear LUT. (c) Complementary image of a hematoxylin and eosin-stained histological section of murine retina. NFL: nerve fiber layer; GCL: ganglion cell layer; IPL: inner plexiform layer; INL: inner nuclear layer; OPL: outer plexiform layer; ONL: outer nuclear layer; IS: inner segment; OS: outer segment; RPE: retinal pigment epithelium; Ch: Choroid; Scl: sclera.

For image processing, the optical path length (OPL) information along the z-axis contained in the B-scans was transformed into geometrical thickness, hereafter thickness *t*, by dividing the OPL by the refractive index of the embedding medium, assuming a value *n*_gel_ = 1.33 (agarose, phytagel) and *n*_resin_ = 1.49 (resin, data provided by manufacturer) for our analysis. Changes in the refractive index at the wavelength of the applied OCT system due to optical dispersion for water-based and polymer-based materials (Δn ≤ 0.02) [61,62] did not induce significant differences to the B-scans within the measuring accuracy of our system (Fig. S1). Subsequently, each B-scan image was filtered with a 3D Gaussian filter (σ*_x_*_,*z* _= 1.0; σ*_y_* = 2.0) (see Fig. 3(a)) and contrast enhancement was applied by linear histogram stretching (see Fig. 3(b)). As a result of this pre-processing of the acquired 3D volume scans, high spatial frequency components like speckle-induced background noise in B-scan images are reduced (for illustration see enlarged areas in Fig. 2(d), Fig. 3(a) and Fig. 3(b)). Moreover, the upper retinal cell layers including the nerve fiber layer (NFL), the ganglion cell layer (GCL), the inner plexiform layer (IPL) and the inner nuclear layer (INL) are clearly resolved (enlarged area in Fig. 3(b)). This allows a confident analysis by image segmentation as described with details below in section 2.3. Figure 3(c) shows a complementary hematoxylin-eosin-stained histological image of a murine retina section that was used as comparison to identify the cell layers that are visible in the acquired B-scans.

### 2.3 Retina layer thickness determination

**Fig. 4.**
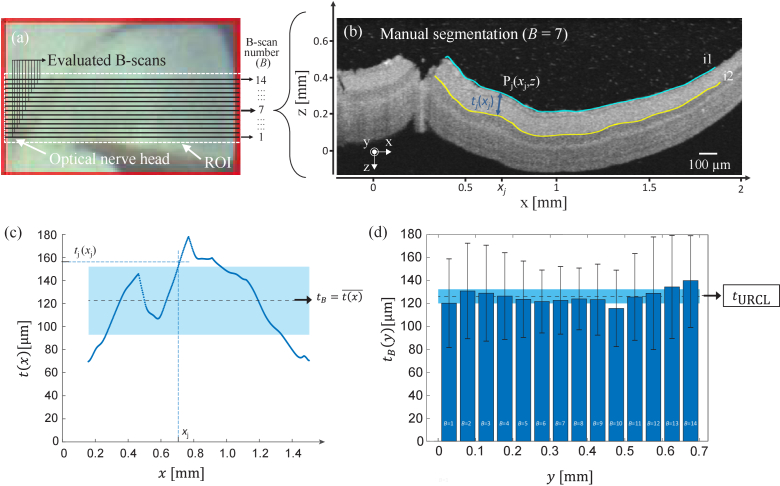
Illustration of the thickness determination of the upper retinal cell layers $t_{\text{URCL}}$ of the developed samples by OCT image segmentation for the example of phytagel-embedded mouse retina prepared by the clover leaf concept (see also Fig. 2(c)–2(d) and Fig. 3(a)–3(b)). (a) Bright field reflected light microscopy image of the volume stack scanned with OCT. The area within the dashed white frame margins the ROI selected for subsequent processing. The black lines within the ROI represent the individual B-scans of a volume stack that were analyzed to determine $t_{\text{URCL}}$ . (b) Segmentation result for the interface between embedding medium and retina *(*i1*,* blue line) and the IPL-INL interface *(*i2*,* yellow line). The thickness of the upper retinal cell layers was determined at the pixel level by calculating the minimal Euclidean distance d from each point P(x,z) of i1 to all points of i2 (the dark blue arrow indicates the minimal distance calculated for an exemplary point P_i_ (*x*,*z*)). (c) Thickness distribution t(x) of the upper retinal cell layers obtained from the B-scan shown in (b). The mean value of the thickness distribution is used to determine the thickness of the URLC for each B-scan ( $t_{B}$ )*.* (d) Distribution of the mean thickness determined for all evaluated B-scans $t_{B}(y)$ that are indicated in (a). The average of the distribution in (d) is used to quantify the thickness of the URCL $t_{\text{URCL}}$ of each fabricated sample. Dashed black lines and light blue horizonal areas in (c) and (d) represent mean value and standard deviation of the depicted distributions, respectively.

For assessment of the generated test-standard structures, the thickness between the embedding medium-retina interface and the IPL-INL interface, from here on denoted as the *upper retinal cell layers* (URCL), of the fabricated samples was determined. This was performed by using custom-built software based on MATLAB scripts that enabled computer-assisted manual segmentation of the cell layers of interest. Firstly, a region of interest (ROI) of B-scans within approximately 1 mm around the optical nerve was preselected from the entire acquired 3D OCT tomogram (e.g., see dashed white rectangle in Fig. 4(a)). Within the preselected ROIs, a set of B-scans (*N*_B-scans_) with a separation of Δ*y* = 50 µm was selected to determine the thickness of the upper retina cell layers $t_{\text{URCL}}$ (see horizontal black lines within the ROI in Fig. 4(a)). Images exhibiting significant retina distortion or folding, e.g., due to preparation artifacts, were discarded from the set of selected B-scans that was subsequently further processed by image segmentation. For each finally selected B-scan, multiple sampling points were selected manually in the OCT image at the embedding medium-retina interface (*i1*) and at the IPL-INL interface (*i2*), see Fig. 4(b). The sampling points were interpolated software assisted by splines to generate continuous lines along the corresponding retina layer interfaces (blue and yellow lines in Fig. 3(b)). Then, for each B-scan, i.e., at each *y*-plane, the thickness distribution t(x) of the URCL was calculated pixelwise for each point P*_j_* (*x*,*z*) in *i1* by calculating the minimum distance: $t_{j}(x)=\min|d({\text{P}}_{\text{j}}(x,z),i2)|$ where *d* is the Euclidean distance (Fig. 4(b)–4(c)) and *j *= 1, …, *m*, where *m* represents the length of *i1* in pixels. Then, the thickness of the URCL for each B-scan $t_{\text{B}}$ of a selected set was calculated by calculating the mean value of the thickness distribution $\bar{t(x)}$ (Fig. 3(d)):

$$t_{B}=\bar{t(x)}=\frac{1}{m}\cdot \overset{m}{\underset{j}{\sum }}t_{j}(x)\;$$

Finally, the thickness of the upper retinal cell layers $t_{\text{URCL}}$ for each sample was determined from the average thickness of all evaluated B-scans:

$$t_{\text{URCL}}=\bar{t_{B}(y)}=\frac{1}{N_{\text{B - scans}}}\cdot \overset{N_{\text{B - scans}}}{\underset{k}{\sum }}t_{k}(y)$$

where *N*_B-scans_ is the number of OCT images evaluated by segmentation (dashed line in Fig. 4(d) and its corresponding standard deviation in light blue).

## 3. Results

The establishment of durable 3D models for OCT performance testing from dissected ex-vivo mouse retina included several steps. First, the impact of different procedures for fixation of the retina tissue were analyzed. Then, the reliability of NMDA for inducing glaucoma-like retinal tissue alterations was validated. Subsequently, different sample preparation methods concerning the retina shape were compared. Finally, the suitability of different embedding media for the fabrication of durable 3D models based on healthy and glaucoma-mimicking mouse retina for OCT imaging was analyzed.

### 3.1 Comparison of different retina tissue fixation procedures and eye treatment with NMDA

**Fig. 5.**
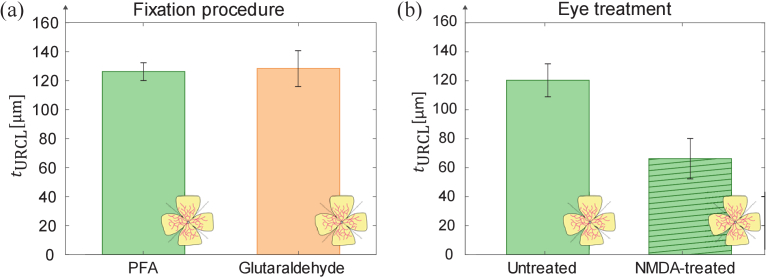
(a) Comparison of the thickness of the upper retinal cell layers from eyes extracted from the same animal (mouse 1) and prepared using different fixation procedures: one fixated in 4% PFA solution in PBS (*N*_B-scans_ = 14) and the other one fixated in 2% glutaraldehyde solution in PBS (*N*_B-scans_ = 21). (b) Comparison of the thickness of the upper retinal cell layers of tissue from eyes extracted from the same animal (mouse 2) which were untreated (*N*_B-scans_ = 21) and injected with NMDA (*N*_B-scans_ = 21). For all measurements in (a)-(b) the retina tissue was prepared using the clover leaf concept. The samples were embedded in (a) phytagel and (b) agarose. Bar diagrams represents average layer thickness values of *N*_B-scans_ evaluated B-scans and error bars indicate the corresponding standard deviation.

In a first comparative study, the impact of two different fixation procedures on the ex-vivo retina tissue was investigated. For this purpose, the thickness of the upper retina layers was measured for two eyes from the same animal (mouse 1) from which one was prepared by fixation in a 4% PFA solution in PBS while the other one was fixated by 2% glutaraldehyde solution in PBS, as described in Section 2.1. Both samples were prepared using the clover leaf concept (Fig. 1(e)) and then embedded in phytagel. The thickness of the upper cell layers of both scanned samples is shown in Fig. 5(a). Using the segmentation approach described in section 2.2, we achieved a thickness for the eye fixated in the PFA solution of the upper retinal cell layers $t_{\text{URCL}}=\;$ 126 ± 6 µm, while a thickness of $t_{\text{URCL}}=\;$ 128 ± 12 µm was measured for the eye fixated in glutaraldehyde. The data indicates no significant differences of the two fixation methods on the retina layer thickness within the measurement uncertainty. As fixation in PFA represents a well-established method for immunohistochemical staining, mice eyes were fixated with 4% PFA solution in PBS for all subsequent experiments.

Secondly, the effect of NMDA to induce degeneration of the RGC in the retina tissue was validated. For this purpose, a solution of NMDA was injected to one eye of a mouse (mouse 2), while the other eye was untreated and served as control. Both eyes were extracted, fixated in 4% PFA in PBS, prepared using the clover leaf concept, and embedded in 1% agarose. The measured thickness for each eye treatment is represented in Fig. 5(b). A thickness of $t_{\text{URCL}}=\;$ 120 ± 11 µm was determined for the untreated eye, while for the NMDA-injected eye a value of $t_{\text{URCL}}=\;$ 66 ± 14 µm was obtained. This represents an almost 2-fold decrease of the upper retina layers thickness which indicates a clear degradation due to NMDA treatment.

### 3.2 Comparison of different specimen shapes and embedding gels

**Fig. 6.**
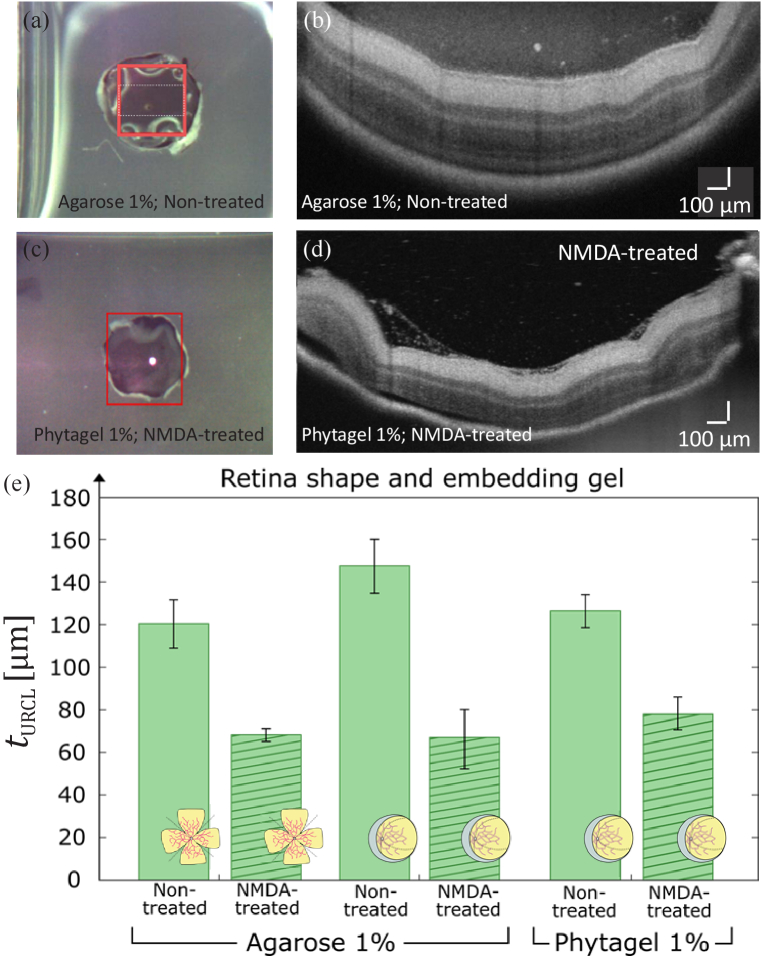
Evaluation of different specimen shapes and embedding gels. (a), (c) Bright-field reflected light microscopy image and (b), (d) representative processed OCT B-scans acquired from mouse retina prepared by eyecup concept for (a)-(b) untreated retina tissue embedded in 1% agarose (mouse 3) and (c)-(d) NMDA-treated retina embedded in 1% phytagel (mouse 4). (e) Thickness *t*_URCL_ of the upper retinal cell layers for untreated and NMDA-treated tissue determined by utilizing different specimen shapes and types of embedding gel: clover leaf concept (mouse 2, data from Fig. 5(b) and eyecup concept (mouse 3, *N*_B-scans_ = 21, data from (a)-(b); mouse 4, *N*_B-scans_ = 21, data from (c)-(d)). Bar diagrams represents average layer thickness values from *N*_B-scans_ evaluated B-scans and error bars indicate the corresponding standard deviation. Eyes employed for the experiments following the eyecup concept were extracted from different animals than for the clover leaf concept. Independently of retina shape and embedding gel, a significantly lower thickness of the upper layers is observed for NMDA-treated retina compared to the corresponding untreated tissue.

To evaluate the suitability of different specimen shapes and embedding gels, the preparation of retina 3D models following the eyecup concept was evaluated and compared with the previous results shown in Fig.5b. For this comparison, untreated and NMDA-treated eyes from two animals (mouse 3 and mouse 4) were fixated utilizing 4% PFA in PBS, prepared using the eyecup concept and embedded in 1% agarose (mouse 3) or in 1% phytagel (mouse 4). Figures 6(a)-(d) show bright-field reflected light microscopy images and corresponding representative B-scan of the acquired OCT volume data set of eyes prepared by the eyecup concept. Figures 6(a)-(b) depict experimental images of untreated retina from mouse 3 embedded in 1% agarose, whereas Fig. 6(c)-(d) show the NMDA-treated retina embedded in 1% phytagel from mouse 4. For the retina tissue embedded in 1% agarose, the measured thickness of the untreated retina was determined to 147 ± 12 µm, while for the NMDA-treated eye the thickness resulted in $t_{\text{URCL}}=\;$ 68 ± 3 µm. For the retina tissue embedded in 1% phytagel, the obtained thickness for the untreated retina was $t_{\text{URCL}}=\;$ 126 ± 20 µm, whereas for the NMDA-treated eye was $t_{\text{URCL}}=\;$ 78 ± 16 µm. The results are compared in Fig. 6(e) together with the data already shown in Fig. 5(b) from mouse 2, which were acquired with the same preparation procedure except that the retina was sectioned by the clover leaf concept. Figure 6(e) shows that in average, for all samples within the measurement uncertainty a considerable thickness decrease of the NMDA-treated retina in comparison to the untreated tissue was determined, although the investigated eyes were extracted from different animals, prepared in different tissue shapes, and were embedded in different gels.

### 3.3 Establishment of long-term stable 3D models of murine retina embedded in resin

To evaluate the fabrication of 3D models with long term stability, we investigated comparatively the suitability of EPON and Gedeo crystal resins as embedding media in the preparation procedure of the retina models. For the characterization of the resulting resin-based 3D models, a set of B-scans were firstly extracted from the acquired OCT volume stacks and compared to the previously acquired image data from phytagel or agarose embedded retina tissue. To quantify light scattering induced by the embedding media, for each sample the standard deviation of the background noise in the pre-processed OCT B-scans was determined in a specified ROI without sample.

**Fig. 7.**
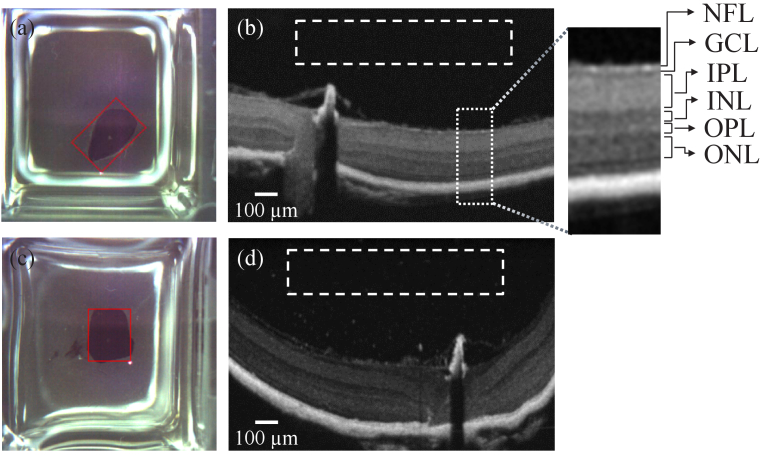
Comparison of different resins for the fabrication of durable 3D models from murine retina tissue from different animals (mouse 5 and mouse 6). (a), (c): Bright-field reflected light microscopy images and (c), (d) corresponding representative pre-processed OCT B-scans from investigations on (a)-(b) EPON and (c)-(d) Gedeo crystal resins as retina embedding media. B-Scans were acquired with the same operation parameters of the OCT system. To quantify the background scattering noise within the B-scans, the standard deviations of the gray levels in specified regions of interest (ROIs) above the sample were determined (see dashed white boxes). The results illustrate that the achieved OCT image quality of 3D tissue samples which are embedded in EPON resin induces slightly lower light scattering than by using Gedeo crystal.

Figure 7(a) and Fig. 7(c) show white light reflected light microscopy images of mouse retina embedded in EPON and Gedeo Crystal resin. Both samples were prepared from untreated eyes from different animals (mouse 5 and mouse 6) following the eyecup dissection concept and, importantly, the same level of resin was prepared on top of both retina tissue. Representative processed B-scans achieved with EPON and Gedeo Crystal resins are illustrated in Fig. 7(b) and 7(d), respectively. Note that, like for the agarose and phytagel embedded retina tissue, the different retinal cell layers can be clearly recognized and distinguished in both cases. This includes also tiny tissue structures of the retina such as GCL, IPL, INL, the outer plexiform layer (OPL), and the outer nuclear layer (ONL). Qualitatively, the image contrast of the resin embedded retina appears to be very similar to the samples prepared in phytagel and in agarose that are depicted in Fig. 6(b) and 6(d), where agarose as embedding medium causes the lowest background image quality.

To further quantify the B-scan quality, the standard deviation of the gray levels in a ROI above the sample was determined (see white dashed boxes in Fig. 7(b), 7(d)). For the EPON sample the standard deviation of the gray level distribution was obtained to $\sigma_{\text{EPON}}$ = 1.44 which is slightly lower that the corresponding value measured for the sample embedded in Gedeo crystal resin ( $\sigma_{\text{Gedeo}}$ = 1.87). For the embedding gels, the background standard deviations in the sample free areas of the images that are shown in Fig.6b and Fig.6d were determined to $\sigma_{\text{agarose}}$ = 10.10 and $\sigma_{\text{phytagel}}$ = 3.18. These values agree with the visual appearance of the background noise in the analyzed B-scans which is higher than for the resin embedded retina. Considering these results, and that EPON is a widespread well characterized mounting medium for electron microscopy and correlative imaging with optical microscopy, we established embedding in EPON as the method of choice for all further investigations on the durable 3D models.

**Fig. 8.**
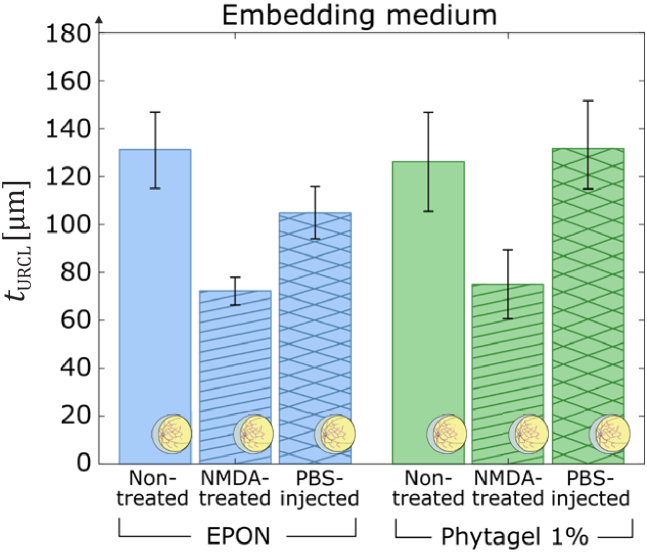
Comparison of 3D retina models fabricated from murine eyes embedded in EPON resin and in phytagel utilizing the eyecup preparation concept for different treatments by determination of the thickness of the upper retinal cell layers. Samples prepared in phytagel were previously fixated in 4% PFA, whereas samples embedded in EPON were previously fixated in a mixture of 2% PFA and 1.25 glutaraldehyde, as described in section 2.1. The obtained URCL’s thickness from non-treated eyes, NMDA-treated eyes and eyes injected with PBS embedded in EPON (blue bars) were $t_{\text{URCL}}=\;$ 132 ± 16 µm (*N*_B-scans_ = 13), 72 ± 6 µm (*N*_B-scans_ = 39), and 104 ± 11 µm (*N*_B-scans_ = 30), respectively, while for retina embedded in 1% phytagel (green bars) the thickness values were determined to $t_{\text{URCL}}=\;$ 126 ± 20 µm (*N*_B-scans_ = 20), 75 ± 14 µm (*N*_B-scans_ = 20), and 132 ± 18 µm (*N*_B-scans_ = 24).

Various retina tissues from different animals (mouse 7, mouse 8 and mouse 9) that were prepared following the eyecup preparation concept, with different treatments and embedded in EPON, were compared with retina tissues prepared with similar treatment but embedded in phytagel (mouse 4 and mouse 10) by determining the URCL thickness $t_{\text{URCL}}$ from the corresponding OCT volume data as illustrated in Fig. 3. Figure 8 shows the measured $t_{\text{URCL}}$ for retina samples from untreated mouse eyes vs. eyes in which NMDA or PBS was injected, and subsequently were embedded in EPON and phytagel. Note that these results reproduce the previous results in Fig. 6 with an almost 2-fold $t_{\text{URCL}}$ decrease of the eyes injected with NMDA with respect to the untreated control. In addition, the value for the mean thickness $t_{\text{URCL}}$ obtained in different embedding media is found similar for both, untreated and NMDA-treated samples. For the PBS-injected eyes, which serve also as a second control in addition to the untreated eyes, the mean thickness of the upper retinal layers of the tissue embedded in phytagel is determined very similar to the untreated sample, suggesting that injection of PBS should not induce alterations in the RGL. However, in the retina tissue from a PBS-injected eye that is embedded in EPON a slight decrease of $t_{\text{URCL}}$ with respect to the untreated case can be observed. Nevertheless, $t_{\text{URCL}}$ is still found significantly higher than for the NMDA-treated eyes, as it can be seen in Fig. 8 from the respective non-overlapping standard deviation error bars.

**Fig. 9.**
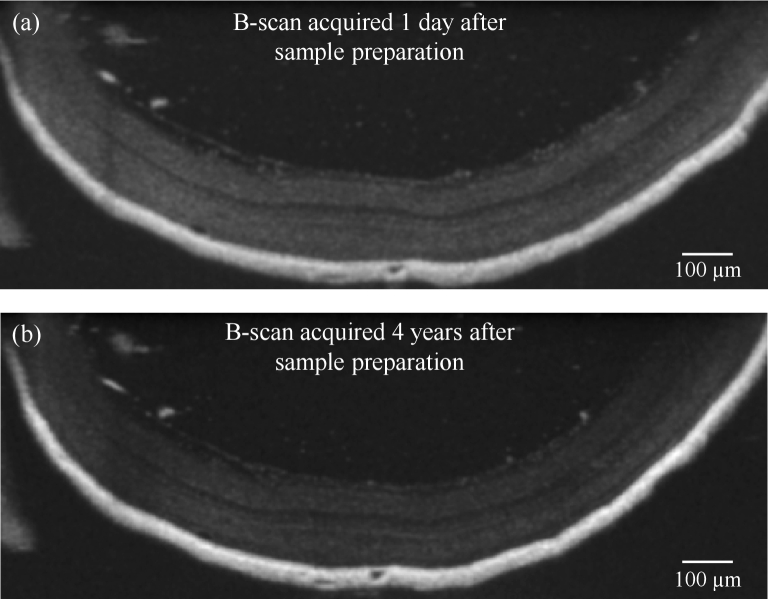
Long-term stability analysis of the established EPON-embedded retina tissue models by comparison of two B-scans from the same 3D retina model (non-treated retina tissue embedded in EPON) acquired (a) 1 day and (b) 4 years after sample preparation.

Finally, the long-term stability of the developed EPON-embedded samples was analyzed. Figure 9 shows in comparison B-scans from OCT volume data acquired at the first day after preparation of the sample and after 4 years. The OCT data were acquired using different versions of the acquisition software (ThorImage version 5.2.0 and version 5.5.7). It can be observed that the retinal morphology and its inner layered structure retained the same morphology without any obvious degradations after this period of time. Minor differences in the shown B-scans are likely due to sample position alignment.

## 4. Discussion and conclusions

Performance characterization of high-resolution OCT systems requires highly stable samples with geometric dimensions and optical properties that match the capabilities and the resolution of the experimental arrangements. Ideally, test standard samples should be durable and robust enough to be easily transported for measurements with systems at different locations [17]. In this proof-of-concept study, we established and analyzed protocols for the preparation of durable 3D models based on healthy and glaucoma-mimicking mouse retina for OCT system performance characterization. The procedure for the preparation of durable 3D models was validated by analysis of the acquired OCT images and the determination of the thickness of the upper retinal cell layers. For the analysis of the prepared 3D models, we considered the depth-of-focus (DOF) of the employed system and embedded the specimen at a depth of twice the system’s DOF in the embedding medium (see Fig. 1(e)-(g)). In this way, we ensured that disturbing reflections from the top and bottom surfaces of the sample do not appear in the OCT images. In addition, by applying a 3-D Gaussian smoothing kernel to the acquired OCT volume data, coherence-induced (speckle-)background noise was significantly reduced during image pre-processing which improved the signal-to-noise ratio of the OCT images (see Fig. 2(d)–3(c)). For the development of the 3D models, we analyzed the influence of different fixation methods (see Fig. 5(a)), validated the chosen animal model as well as the procedure to induce glaucoma-like retina alterations (see Fig. 5(b)), discussed different retina sectioning procedures (see Fig. 6), and investigated the usage of different media for embedding of the retina tissue (Fig. 6–8).

We first analyzed murine retina tissue fixated in PFA and glutaraldehyde, which are chemical fixatives widely used in laboratories for immunohistochemistry [63]. Our investigation on the influence of the fixation method showed that no significant differences on the thickness of the upper retinal cell layers were found in the 3D models fabricated using the investigated fixatives (Fig. 5(a)). In addition, in both cases the cell layers of the retina samples were clearly resolved by the employed OCT system. However, it has been reported that these popular fixation methods have yet some drawbacks, such as an increase of background signals, like autofluorescence, in other microscopy techniques at high concentrations for glutaraldehyde, and morphological disruption produced by slow fixation and cytotoxic effects in the case of PFA [63]. In this context, the dilution of glutaraldehyde with PFA combines the advantages of each fixative and allows to overcome these drawbacks. Therefore, we further investigated a mixture of PFA and glutaraldehyde as the fixation method and, finally, we established this strategy for the fabrication of our 3D retinal models embedded in resin. For the three employed fixation procedures, the OCT image quality and reproduction of retinal layer structures in B-scans results were found in well agreement with image data from earlier in vivo investigations on the same mouse model [64]. In contrast to the similar OCT image quality, the absolute retina thickness (Fig.S1) was determined with lower values than for reported data from in vivo investigations [64], which was also observed in the B-Scan images of agarose and phytagel embedded samples. This effect may be explained by tissue shrinking effects due to the fixing protocols [65].

Note that in the determination of the absolute thickness, due to the applied software the sample refractive index distribution could not be considered in the calculation of the geometric thickness from the acquired OPL data. Regarding this issue, more sophisticated OCT image analysis algorithms might provide more accurate evaluation of retinal cell layers [66]. In addition, it is important to consider optical dispersion in the determination of the retina thickness. However, recent studies suggest no significant variation of the retina refractive index within a similar wavelength range [67], suggesting together with the results shown in Fig.S1, that optical dispersion does not significantly impact the results in our study.

In order to fabricate 3D models from mouse retina with glaucoma-like alterations, we employed C57BL/6J mice as animal model and injected a solution of NMDA to induce degeneration of the upper retinal cell layers. OCT investigations with subsequent segmentation performed on retina tissue from healthy and NMDA-treated mice eyes for various sample preparation procedures demonstrated a 2-fold decrease in the retinal thickness for all applied embedding media (Fig. 6(e) and Fig. 8). The observed degeneration of the upper retina layers for NMDA-treated eyes agrees with results from various earlier studies in which NMDA-treatment is reported to induce glaucoma-like retina alterations, promoting its use for the fabrication of glaucoma-simulating test standards [68–70].

Regarding retina shape, our results demonstrate that both applied sectioning procedures, denoted as in our study clover leaf and eyecup concept, are applicable to achieve acceptable OCT images from untreated retina and NMDA-treated animals (Fig. 4(b) and Fig. 6(b), 6(d)). In practice, due to the cutting procedures, we observed that the clover leaf concept caused increase mechanical damages in the retina compared with the concept in which the eyecup was preserved. Moreover, the eyecup concept provided a high stability of the retina sample which resulted in a higher preservation of the natural shape of the mouse eye during the further preparation steps. Thus, we focused the final development of the 3D retina models on the eyecup concept.

A critical issue for acquiring high quality OCT images is to use an embedding medium that is transparent in the OCT light wavelength range and features a low turbidity to avoid undesired light scattering. Therefore, we evaluated the suitability of various embedding media for the retina tissue for a light wavelength around 905 nm. Phytagel and agarose were used initially as transparent and natural embedding media with physiological osmolarity to preserve retina cell layers similar to native tissue. To accomplish 3D models with high temporal stability, we investigated EPON and Gedeo crystal resins as embedding media of the retina tissue. The developed samples featured layered structures like native tissue from mouse retina, similarly to the samples embedded in agarose and phytagel, including cell layers with thicknesses close to the OCT resolution limits of the employed commercial HR research OCT system (Fig. 7).

Among the investigated media, the lowest background scattering was detected for resin-based samples, where slightly lower scattering was achieved with EPON than with Gedeo crystal resin, while agarose-embedded samples induced the highest background noise level. Considering these findings, as well as that EPON is a well-known mounting resin for electron microscopy and correlative imaging with optical microscopy, EPON was chosen as embedding medium in the final development of durable 3D models from healthy and glaucoma-mimicking mouse retina. Finally, artificially generated glaucoma-like alterations in the upper retinal cell layers of NMDA-treated mouse eyes could be reliably generated and detected for both, tissue embedded in physiological media as well as in EPON (Fig. 8). Importantly, samples prepared in agarose degenerated after several weeks, whereas for the corresponding resin embedded tissues no changes of their characteristics were observed for a period of 4 years (Fig. 9). This allowed in a recent reported study the comparison of different HR OCT systems in various laboratories with the same sample [71]. Moreover, results from this earlier report validated the applicability of our 3D models for usage with systems operating at a central wavelength of 1370 nm and demonstrated their prospect for performance characterization of OCT system, e.g., during development.

Future applications of our established retina models require a standardization. Standardization of the models is crucial for the broad use for OCT performance testing and calibration. In general, tissue models from living animals are heterogenous and limited in standardization. The standardization has to cover the development and use of standard operation procedures (SOP) for reproducible tissue model production and continuous testing. This includes investigations on parameters such as temperature- or age-dependent alterations of the resin and the embedded tissues as well as fixing procedures, to mention a few, which will contribute to a reliable standardization of retina tissues for OCT measurements.

In conclusion, we have generated durable 3D retina tissue phantoms from healthy eyes and an in vivo glaucoma model that reflect the complexity of murine eyes. We established 3D tissue models applicable for OCT imaging from mouse retina by means of appropriate embedding protocols with preserved optical and morphological properties similar to healthy and diseased tissues. The durable samples developed in this proof-of-concept study feature layered structures with optical properties that are suitable for performance characterization of HR OCT systems with capabilities for detection of alterations in thin layered structures, e.g., as required for improved glaucoma diagnosis. The good agreement with results from previously reported in in vivo OCT studies supports the practical relevance of our approach. The evaluated resin-based sample embedding concept also represents a basis for follow up studies, that together with more sophisticated quality assessment and segmentation methods [72–74], prospect to gain further insights into the underlying mechanisms of the embedding process and accompanied changes of the optical sample properties as well as for the further optimization and standardization of biological tissue-based 3D models for performance evaluation of label-free optical imaging techniques. In summary, the phantoms prospect applications in OCT development, performance characterization and calibration.

## Data Availability

Data underlying the results presented in this paper are not publicly available at this time but may be obtained from the authors upon reasonable request.

## Supplemental document

See Supplement 1 for supporting content.
