# Supplementary material for: Durable 3D murine ex vivo retina glaucoma models for optical coherence tomography
Source: Biomed Opt Express. 2023 Aug 2;14(9):4421–38. doi: 10.1364/BOE.494271 (PMC10545187; doi:10.1364/BOE.494271)
Supplement: Supplementary file 1 [file boe-14-9-4421-s001.pdf]

Supplemental Document

Biomedical Optics EXPRESS

Durable 3D murine ex vivo retina glaucoma models for optical coherence tomography: supplement

ÁLVARO BARROSO,[1,*] STEFFI KETELHUT,[1] GERBURG NETTELS-HACKERT,[2] PETER HEIDUSCHKA,[2] ROCÍO DEL AMOR,[3] VALERY NARANJO,[3] BJÖRN KEMPER,[1,†] AND JÜRGEN SCHNEKENBURGER[1,†]

[1]*Biomedical Technology Center of the Medical Faculty, University of Muenster, Mendelstr. 17, D-48149 Muenster, Germany*
[2]*Department of Ophthalmology of the Medical Faculty, University of Muenster, Domagkstr. 15, D-48149 Muenster, Germany*
[3]*Instituto Universitario de Investigación en Tecnología Centrada en el Ser Humano, Universitat Politècnica de València, Valencia, Spain*
[†]*equally contributing authors*
[*]*alvaro.barroso@uni-muenster.de*

This supplement published with Optica Publishing Group on 2 August 2023 by The Authors under the terms of the Creative Commons Attribution 4.0 License in the format provided by the authors and unedited. Further distribution of this work must maintain attribution to the author(s) and the published article's title, journal citation, and DOI.

Supplement DOI: https://doi.org/10.6084/m9.figshare.23593548

Parent Article DOI: https://doi.org/10.1364/BOE.494271

Durable 3D murine ex vivo retina glaucoma model for optical coherence tomography

ÁLVARO BARROSO,[1,*] STEFFI KETELHUT,[1] GERBURG NETTELS-HACKERT,[2] PETER HEIDUSCHKA,[2] ROCÍO DEL AMOR,[3] VALERY NARANJO,[3] BJÖRN KEMPER,[1,+] AND JÜRGEN SCHNEKENBURGER[+]

[1]*Biomedical Technology Center of the Medical Faculty, University of Muenster, Mendelstr. 17, D-48149 Muenster, Germany*
[2]*Department of Ophthalmology of the Medical Faculty, University of Muenster, Domagkstr. 15, D-48149 Muenster, Germany*
[3]*Instituto Universitario de Investigación en Tecnología Centrada en el Ser Humano, Universitat Politècnica de València, Valencia, Spain*
+equally contributing authors
**alvaro.barroso@uni-muenster.de*

Supplementary information

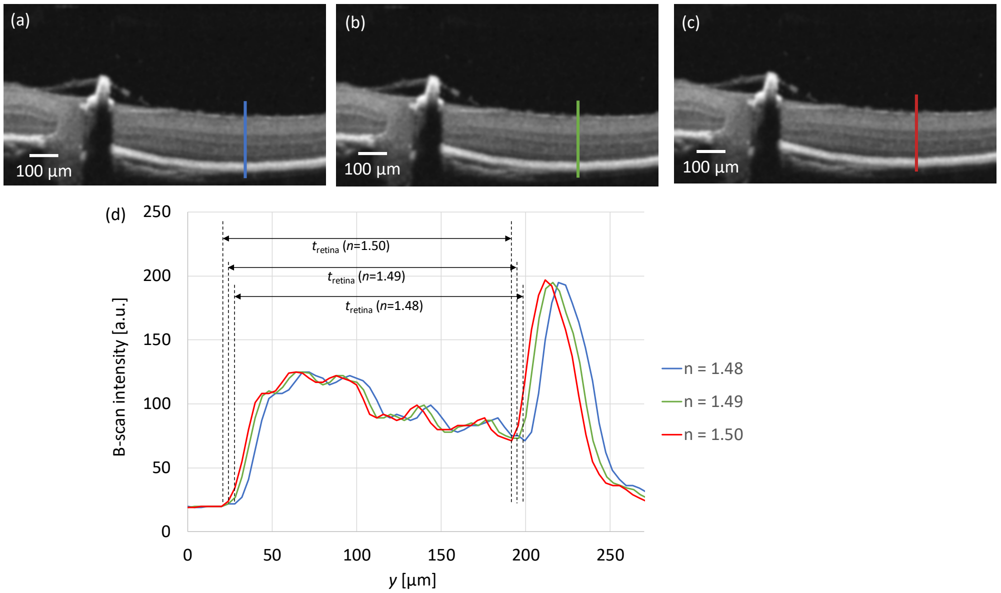

Fig. S1: Analysis of the possible impact of refractive index variations of the embedding media on the retina thickness determination. (a)-(c) Generated B-scans in which the geometric dimensions of the sample were determined by considering a embedding medium's refractive index of n = 1.48, 1.49, and 1:50, respectively. (d) Intensity of the B-scan images across the indicated lines in (a)-(c). The cross-section plots illustrate that the intensity of the B-scans is mainly shifted along the cross-section direction. The thickness of the retina t_{retina} obtained from the cross sections was determined to t_{retina} = 163.6 µm, 163.9 µm and 163.6 µm ± 4.0 µm, which is close to maximal axial resolution of the employed OCT system.
